# Enfuvirtide in therapy at patients with HIV-infection and tuberculosis

**DOI:** 10.1186/1758-2652-13-S4-P197

**Published:** 2010-11-08

**Authors:** AV Kravchenko, AV Kuznetsova, OA Kozyrev, VA Lukyanova, VG Kanestri

**Affiliations:** 1 Russia Federal AIDS Center, Moscow, Russian Federation; 2 Khabarovsk regional AIDS Center, Khabarovsk, Russian Federation; 3 Volgograd regional AIDS Center, Volgograd, Russian Federation; 4 Altay regional AIDS Center, Barnaul, Russian Federation

## Background

The certain difficulties in a choice of HAART regime arise at presence at the patient with HIV-infection and tuberculosis. Enfuvirtide (ENF) not metabolized by enzymes of system cytochrome Ð450. It does not cooperate with NNRTI, PI and rifamycins and can be added to HAART regime if the patient to accept antimycobacterial drugs.

## Purpose of the study

Comparison of efficiency and safety of HAART regimes with and without ENF in patients with HIV-infection and tuberculosis.

## Methods

This research was not randomized and was lead in conditions of real clinical practice. 81 pts with HIV-infection and tuberculosis were divided on 2 groups: 1 gr. - 26 pt. with the standard regimes of HAART (2 NRTI + PI/r or NNRTI) and ENF; 2 gr. - 55 pts with the standard regimes of HAART. We determined clinical symptoms, CD4+ lymphocyte's count, viral load, haematological and biochemical parameters before, 4, 12 and 24 weeks therapy.

## Summary of results

Within 24 weeks HAART the survival rate of patients of 1 gr. has made 96,2%, and 2nd - 89,1%. After 24 weeks HAART the share of pts with VL <500 copies/ml at pts 1 gr. was 81,8% and 2 gr. - 65% (OT- analysis, p <0,05) and 76% - 50% (ITT-analysis, p <0,05). After 24 weeks HAART the median of a gain of quantity CD4+ lymphocytes at pts of 1 gr. has made +110 cells, and 2 gr. - 69 cells (p <0,05). Safety of both regimes of HAART was quite good. Only 1 pt after 12 weeks of treatment has refused therapy with ENF because of local reactions.

## Conclusions

Inclusion in regimes of HAART of ENF promoted increase in a share of pts with not determined HIV RNA level and to more essential growth of CD4+lymphocyte's count. Addition to standard regime of HAART of ENF essentially did not influence on the frequency of the clinical or laboratory adverse events caused by treatment.

